# Supplementary material for: Impaired Kidney Function Associated with Increased Risk of Side Effects in Patients with Small Vessel Vasculitis Treated with Rituximab as an Induction Therapy
Source: J Clin Med. 2021 Feb 16;10(4):786. doi: 10.3390/jcm10040786 (PMC7920022; doi:10.3390/jcm10040786)
Supplement: Supplementary file 1 [file jcm-10-00786-s001.pdf]

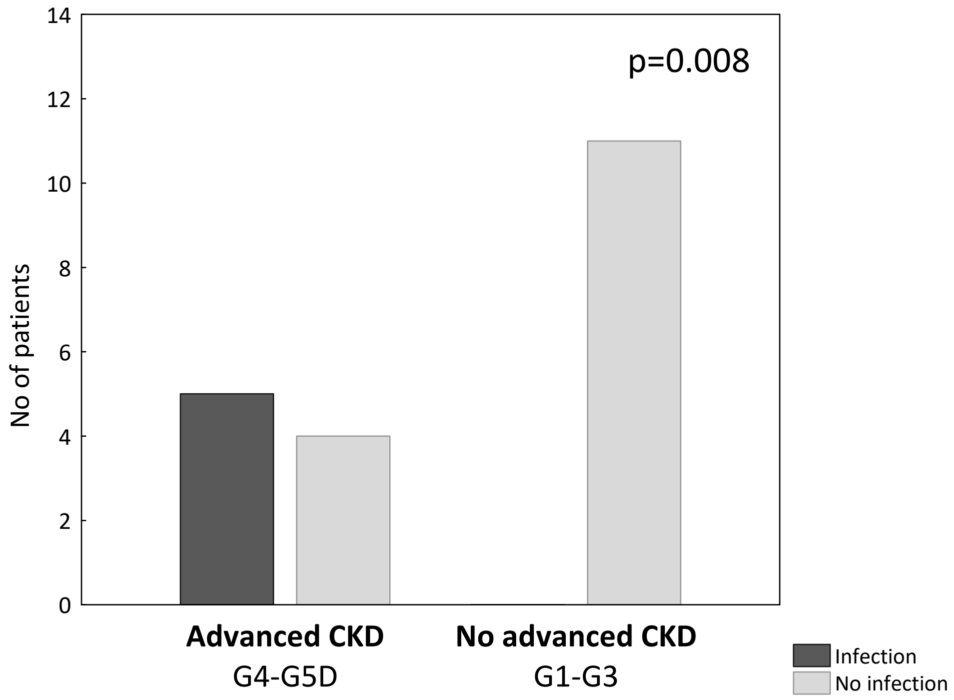

Figure 1. Infections in patients with and without advanced CKD treated with rituximab.

Table S1. Comparison of SVV patients with and without advanced CKD.

**Parameter**	**Average values**	**Advanced CKD** n=9	**No advanced CKD** *n=11*	**p-value**
Age [years]	mean±SD	51±16	45±11	0.308*
Sex:<br>Male<br>Female	 n (%)<br>n (%)	 4 (44%)<br>5 (56%)	 6 (55%)<br>6 (45%)	1.000#
ANCA type:<br>PR3-ANCA<br>MPO-ANCA<br>ANCA negative	 n (%)<br>n (%)<br>n (%)	 8 (89%)<br>0<br>1 (11%)	 10 (91%)<br>1 (9%)<br>0	0.360#
Duration of SVV [years]	median [IQR]	2 [1-5]	3 [3-11]	0.296
CYC cumulative dose [g]	median [IQR]	9 [7.5-13.5]	13 [8.3-40]	0.470
CYC cumulative dose [mg/kg]	median [IQR]	105 [87-262]	157 [129-430]	0.447

RTX single dose [mg]	mean±SD	690±130	731±80	0.390
Baseline BVAS/WG score	median [IQR]	7 [7-9]	5 [4-12]	0.125
SVV course:<br>- limited or severe flare<br>- severe multiorgan flare	 n (%)<br>n (%)	 5 (56%)<br>4 (44%)	 10 (91%)<br>1 (9%)	0.069
Dominant organ activity (presence of major symptoms):<br>- eye<br>- ENT<br>- pulmonary<br>- gastrointestinal<br>- renal<br>- nervous system<br>- other	 n (%)<br>n (%)<br>n (%)<br>n (%)<br>n (%)<br>n (%)<br>n (%)	 1<br>1<br>2<br>0<br>4<br>1<br>2	 0<br>3<br>0<br>1<br>0<br>2<br>2	-
Baseline ANCA titre [IU/mL]	median [IQR]	21 [2-102]	47 [10-124]	0.408
RTX efficacy:<br>- complete remission<br>- partial remission<br>- inadequate response	 n (%)<br>n (%)<br>n (%)	 9 (100%)<br>0<br>0	 8 (73%)<br>2 (18%)<br>1 (9%)	0.236

*P-values for Mann-Whitney's test, *p-value for t-test, #p-value for chi-square test.*
